# Structure-guided engineering of an aromatic ring-hydroxylating dioxygenase for broad-spectrum phthalate degradation

**DOI:** 10.1128/jb.00221-25

**Published:** 2025-08-12

**Authors:** Jai Krishna Mahto, Ishani Mishra, Kuldeep Jangid, Pravindra Kumar

**Affiliations:** 1 Department of Biosciences and Bioengineering, IIT Roorkee 30112, Roorkee, India; Department of Microbiology and Cell Science, University of Florida, Gainesville, Florida, USA

**Keywords:** isophthalate dioxygenase, Rieske oxygenase, mononuclear iron, enzyme engineering, mutagenesis, isophthalate, phthalate, terephthalate

## Abstract

Phthalates such as isophthalate, phthalate, and terephthalate are widespread environmental pollutants with significant health and ecological impacts. *Comamonas testosteroni* KF1 initiates isophthalate degradation through a specialized two-component enzyme system composed of isophthalate dioxygenase (IPDO) and its cognate reductase, isophthalate dioxygenase reductase. Despite its environmental significance, the lack of structural insights into IPDO has hindered efforts to rationally redesign, optimize, and harness its chemistry. Here, we report the first crystal structures of substrate-free IPDO and its complex with isophthalate, revealing unique structural features that underpin its substrate specificity. Unlike related oxygenases, phthalate dioxygenase (α_3_α_3_) and terephthalate dioxygenase (α_3_β_3_), IPDO adopts a trimer (α_3_) architecture, with distinct active site residues tailored to isophthalate binding. The comparative structural analysis identified steric and electrostatic constraints—particularly involving residue V178—that preclude the binding of ortho- or para-substituted substrates. Leveraging these structural insights, we engineered IPDO variants with broadened substrate specificity. Notably, the V178A and F249H substitutions enabled the enzyme to degrade three regioisomers of phthalate (phthalate, isophthalate, and terephthalate) without diminishing its native activity against isophthalate. The catalytic turnover (*k*_cat_) of the V178A/F249H double mutant was found to be 4.8 ± 0.3, 4.9 ± 0.2, and 4.0 ± 0.2 s^−1^ for isophthalate, terephthalate, and phthalate, respectively, demonstrating comparable catalytic efficiency for all three substrates. Overall, this work advances our understanding of the molecular mechanisms involved in isophthalate dihydroxylation and elucidates a rational engineering approach to expand the catalytic repertoire of IPDO for biotechnological and environmental applications.

**IMPORTANCE:**

Phthalate pollution poses a major environmental concern due to its widespread use as plasticizers and its persistence in ecosystems. Microbial degradation of phthalates offers a sustainable solution for mitigating this contamination. Among the key enzymes involved, aromatic-ring-hydroxylating dioxygenases initiate the first critical step in phthalate breakdown. However, most known enzymes exhibit narrow substrate specificity, limiting their utility for degrading diverse phthalate isomers such as isophthalate, phthalate, and terephthalate. This research addresses a critical gap by elucidating the structural basis of substrate specificity in isophthalate dioxygenase and applying rational engineering to expand its catalytic range. By generating enzyme variants capable of degrading all three phthalate regioisomers, this work provides a blueprint for designing versatile biocatalysts tailored for pollutant detoxification.

## INTRODUCTION

Phthalates and their esters are hazardous compounds commonly incorporated into plastics to enhance flexibility and durability (1–3). Among them, terephthalate, a key building block of polyethylene terephthalate plastics, further contributes to the global plastic burden (4). Owing to their extensive usage, phthalates leach into the environment, raising critical ecological and public health concerns (5–8). Numerous studies have demonstrated their role as endocrine disruptors, linked to reproductive toxicity, neurodevelopmental disorders, and carcinogenesis (2, 9–12). Consequently, the efficient degradation of all three phthalate regioisomers—phthalate, isophthalate, and terephthalate—is a pressing priority.

Among known bacterial pathways, the isophthalate degradation route in *Comamonas testosteroni* is one of the few that can selectively transform isophthalate, an understudied isomer, via the *iphA2CBA1* gene cluster (1, 13). This cluster encodes isophthalate dioxygenase (IPDO), along with its partner reductase and associated transporter and dehydrogenase proteins. IPDO, a Rieske oxygenase (RO), catalyzes the initial and critical dihydroxylation of isophthalate, producing *cis*-3,4-diol isophthalate, thereby priming the molecule for further catabolism (1, 13).

Rieske oxygenases form a vast enzyme class—comprising over 70,000 members—known for their chemo-, regio-, and stereoselective oxidative transformations across diverse substrates (14). These enzymes catalyze mono- and dioxygenation reactions through a conserved catalytic scaffold consisting of a Rieske [2Fe-2S] cluster and a mononuclear non-heme iron center (15–21). The most extensively characterized ROs catalyze the dihydroxylation of aromatic compounds, such as naphthalene (18, 22–24), phthalate (25–28), and biphenyl (19, 29–31). While the general catalytic cycle is increasingly well understood, structural and mechanistic determinants guiding substrate scope and oxidation outcomes remain elusive for many ROs (32, 33). This is particularly true for isophthalate degradation, where no structure of IPDO has been reported to date.

By contrast, homologous ROs such as phthalate dioxygenase (PDO) (3) and terephthalate dioxygenase (4, 34) have been structurally characterized, enabling structure-guided engineering to expand substrate scope or modulate regioselectivity. These advances—especially in engineering enzymes like naphthalene dioxygenase and biphenyl dioxygenase to oxidize a wider range of polychlorinated biphenyls—demonstrate the potential of RO reprogramming to address persistent organic pollutants (35–40). Yet, IPDO remains an untapped candidate for such efforts, despite its natural specificity for isophthalate, a key environmental contaminant.

In this study, we present the first crystal structure of IPDO from *C. testosteroni* KF1 (IPDO_KF1_), both in its apo form and in complex with isophthalate. Structural comparisons with other ROs reveal unique features in the active site pocket and substrate recognition elements that likely contribute to IPDO_KF1_’s exclusive specificity. By conducting structure-guided mutagenesis and activity assays, we identify residues essential for substrate positioning. Furthermore, we engineer an IPDO_KF1_ variant capable of accepting alternate phthalate regioisomers, thereby establishing a foundation for reprogramming RO enzymes to perform divergent oxidative transformations. These findings not only elucidate the structural basis for isophthalate recognition but also open avenues for rational engineering of Rieske oxygenases to target a broader spectrum of synthetic pollutants.

## RESULTS

### The overall structure of IPDO_KF1_

**Fig 1:**
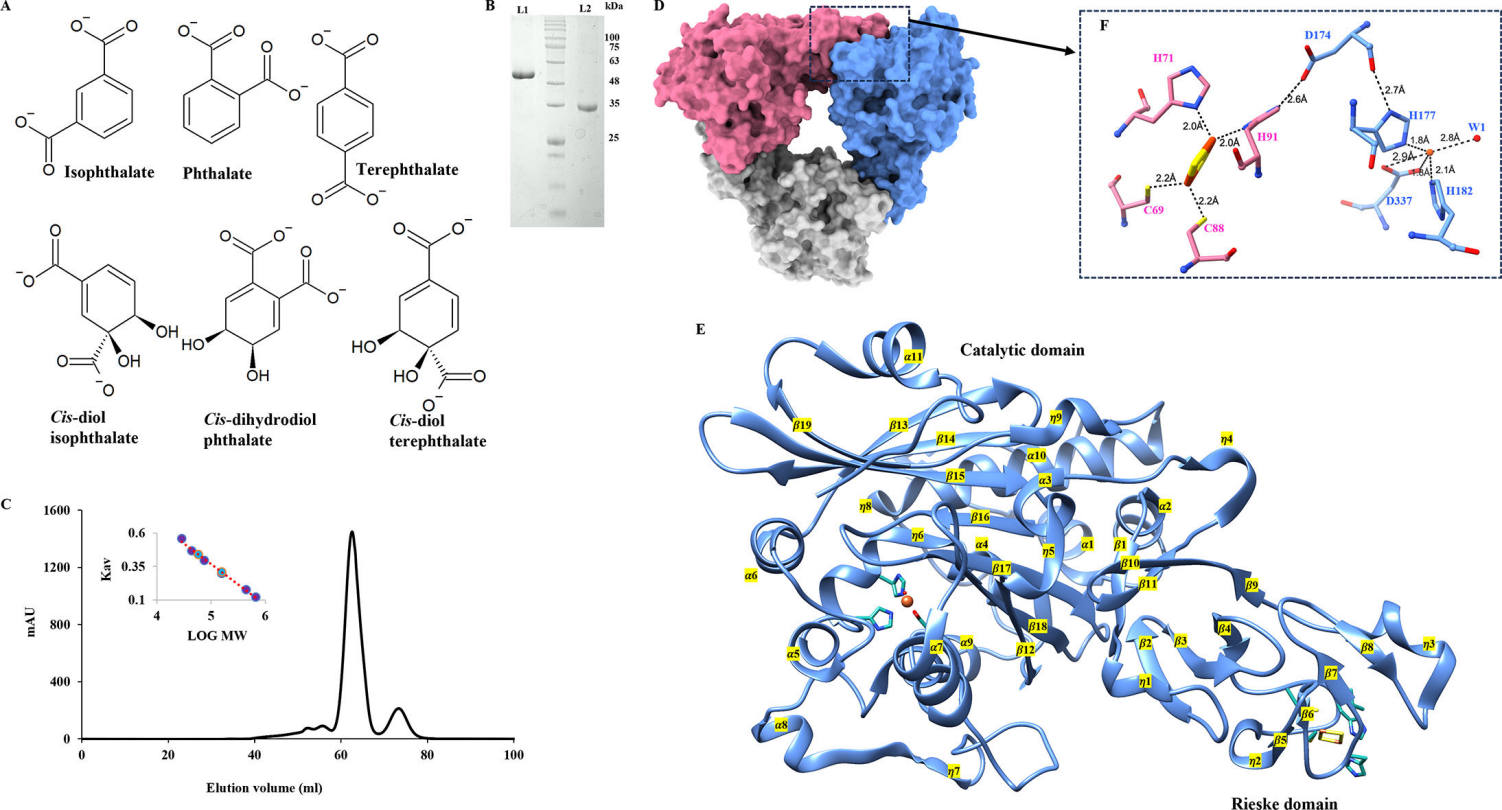
Purification and crystal structure of IPDO_KF1_. (**A**) Chemical structures of isophthalate, phthalate, and terephthalate, along with their corresponding dihydroxylated products formed by dioxygenases: *cis*-diol isophthalate, *cis*-dihydrodiol phthalate, and *cis*-diol terephthalate. (**B**) SDS-PAGE analysis of (L1) IPDO_KF1_ purified fraction (~50 kDa) and (L2) IPDR_KF1_ (~34 kDa) purified fraction using Ni-NTA chromatography. (**C**) The elution profiles of IPDO_KF1_ by gel filtration chromatography using a Superdex 200 column. The chromatogram shows that IPDO_KF1_ elutes at 62 mL corresponding to the molecular mass of IPDO_KF1_ trimer (~150 kDa) and at 73 mL corresponding to the molecular mass of IPDO_KF1_ monomer (~50 kDa). (**D**) Cartoon representation of IPDO_KF1_ α_3_ trimer; subunits are colored in gray, blue, and pink. (**E**) Cartoon representation of an IPDO_KF1_ protomer with secondary structure elements. (**F**) Residue D174 serves as a bridge between the Rieske center to the mononuclear iron center. The coordination of the mononuclear iron center with residues and a water molecule, as well as the Rieske center and its interacting residues, is shown.

To enable crystallization, the IPDO_KF1_ enzyme was heterologously expressed in *Escherichia coli* BL21 cells and subsequently purified (Fig. 1B and C). The apo-form crystal structure of IPDO_KF1_ was determined to a resolution of 2.96 Å through molecular replacement phasing using the AlphaFold (41) predicted model as the search model. The crystals were found to belong to the space group *P* 2_1_ 3 and contained a single monomer per asymmetric unit. Comprehensive data collection and refinement statistics, along with stereochemical parameters, are summarized in Table 1. The crystallographic symmetry supported a trimeric architecture (α_3_ arrangement), where each protomer was related by a non-crystallographic threefold axis (Fig. 1D). Structurally, each IPDO_KF1_ protomer comprises 424 amino acids and adopts a fold typical of other ROs, comprising a Rieske domain and a catalytic domain as (3, 15–17, 21, 32–34) (Fig. 1E).

**TABLE 1:** Data processing and refinement statistics^*e*^

	IPDO_KF1_	IPDO_KF1_-isophthalate
Data collection		
Resolution range	61.54–2.96 (3.07–2.96)^*a*^	25.22–3.30 (3.42–3.30)
Unit cell dimensions a, b, c (Å)	150.74, 150.74, 150.74	146.96, 146.96, 146.96
Space group	*P* 2_1_ 3	*P* 2_1_ 3
Completeness (%)	99.96 (100.00)	99.41 (99.81)
*R*_merge_ (%)^*b*^	3.5 (29.6)	38.8 (77.6)
*R*_pim_ (%)	3.5 (29.6)	26.0 (58.9)
CC (1/2)	0.99 (0.81)	0.63 (0.16)
I/σ(I)	15.0 (2.4)	9.9 (2.5)
Wilson B-factor (Å^2^)	77.78	64.75
Refinement		
Reflections used in refinement	23,984	16,084
Reflections used for *R*_free_	1,205	769
*R*_work_^*c*^	0.180	0.182
*R*_free_^*c*^	0.207	0.225
Number of non-hydrogen atoms	3,526	3,484
Macromolecules	3,514	3,467
Ligands	5	17
Solvent	7	
Protein residues	440	435
RMS (bonds) (Å)^*d*^	0.010	0.010
RMS (angles) (°)^*d*^	2.05	2.12
Favored (%)	94.29	89.84
Allowed (%)	5.48	9.70
Outliers (%)	0.23	0.46
Average B-factor (Å^2^)	78.33	67.78
Macromolecules (Å^2^)	78.43	67.84
Ligands (Å^2^)	62.38	56.84
Solvent (Å^2^)	36.32	

^*a*^ Values in parentheses are for the highest resolution shell.

^*b*^ *R*_merge_ = Σ| *I* − 〈*I*〉|/Σ*I.*

^*c*^ *R* = Σ|*F*_obs_| − |*F*_calc_|/Σ|*F*_obs_|. The *R*_free_ is the *R* calculated on the 5% reflections excluded for refinement.

^*d*^ RMS is root mean square.

^*e*^ Empty cell indicates that there is no solvent in the IPDO_KF1_-isophthalate complex structure.

The Rieske domain, spanning residues 25–144, includes four η-helices (η1–η4) and three β-sheets (β1–β11) with three to four β-strands each. Its Rieske center, the iron-sulfur cluster, is coordinated by C69 and C88 to Fe1 at a distance of 2.2 Å each, and by H71 and H91 to Fe2 at a distance of 2.0 Å each, with two bridging sulfide ions stabilizing the cluster (Fig. 1F). The catalytic domain features an eight-stranded (β12–β19) antiparallel β-sheet surrounded by 11 α-helices (α1–α11) and 5 η-helices (η5–η9). The mononuclear iron atom at the active site is coordinated to H177 and H182 at 1.8 and 2.1 Å distances, respectively, as well as to D337 with 1.8 Å distance (Fig. 1F). Additionally, a solvent molecule is also bound to the mononuclear iron at 2.8 Å distance (Fig. 1F). Notably, while the Rieske center and the mononuclear iron are 45 Å apart within the same protomer, their inter-subunit distance is only 12.5 Å, consistent with electron transfer occurring between subunits in RO enzymes. The residue D174, located at the protomer interface, serves as a bridge connecting the two metal centers (Fig. 1F), consistent with the structural organization observed in other ROs (22, 42).

**Fig 2:**
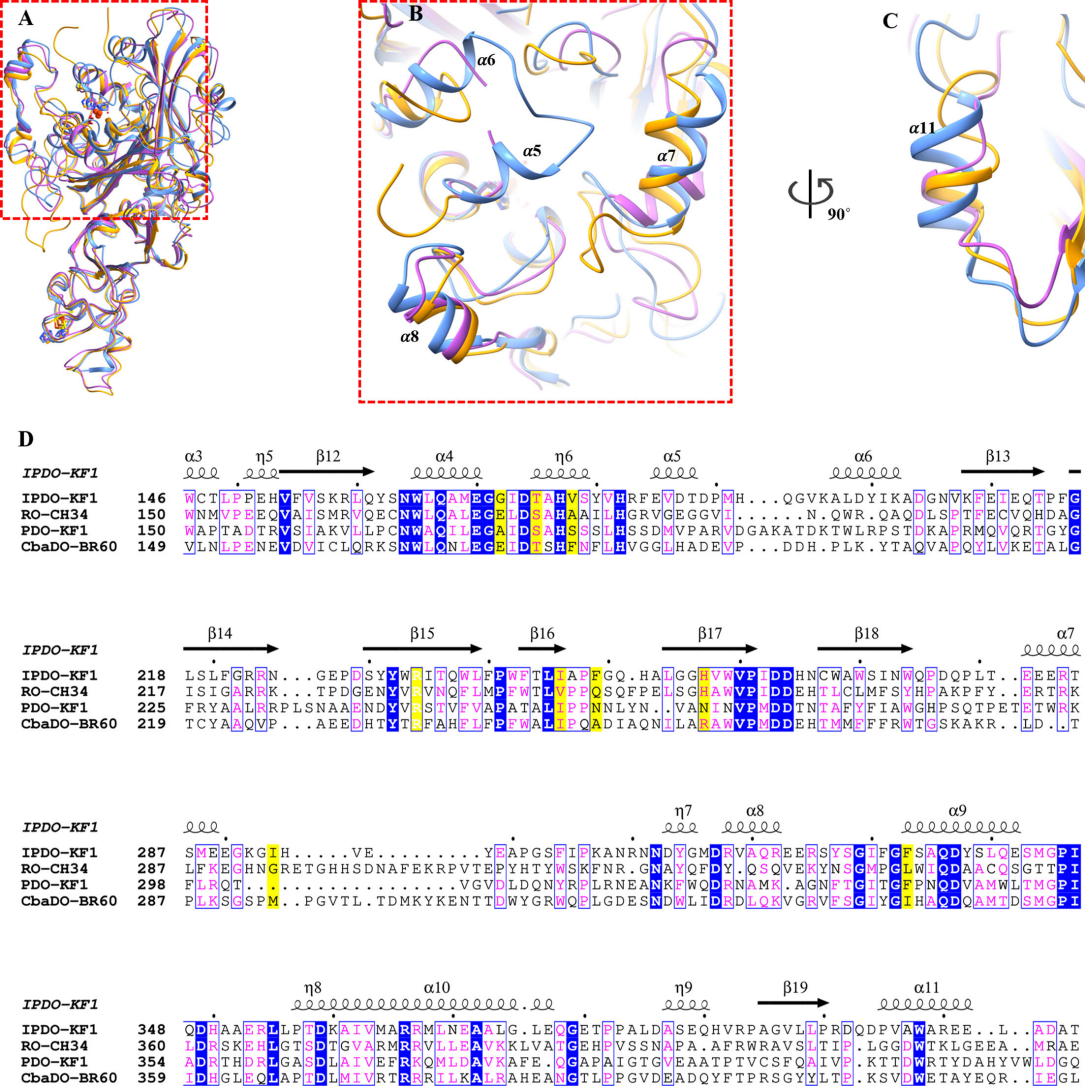
Structural and sequence comparison of IPDO_KF1_ with its homolog ROs. (**A**) The IPDO_KF1_ structure is superposed with its closest structural homologs. A cartoon representation of IPDO_KF1_ (blue), PDO_KF1_ (magenta), and RO_CH34_ (orange) is shown. (**B**) The IPDO_KF1_ structure shows two helices (α5 and α6) that cover the active site; the corresponding region is absent in PDO_KF1_ and RO_CH34_ structures. Few other α-helices of IPDO_KF1_, such as α7, α8, and (**C**) α11, are seen to not align with its homologs. (**D**) Amino acid sequence alignment of IPDO_KF1_ with related ROs. The aligned sequences are IPDO from *Comamonas testosteroni* KF1 (IPDO-KF1), Rieske oxygenase from *Cupriavidus metallidurans* CH34 (RO-CH34), phthalate dioxygenase from *Comamonas testosteroni* KF1 (PDO-KF1), and 3-chlorobenzoate dioxygenase from *Alcaligenes* sp. BR60 (CbaDO-BR60) (43). Identical residues have a blue background. Similar residues are highlighted with blue boxes. The yellow background highlights residues that interact with the substrate. The program ESPript was used for visualization.

Comparative structural analysis using the DALI server showed that IPDO_KF1_ retains the general architecture typical of ROs (Fig. 2A), sharing structural homology with RO_CH34_ (RMSD = 1.8 Å, 38% sequence identity) and PDO_KF1_ (RMSD = 2.0 Å, 31% sequence identity). Despite this similarity, IPDO_KF1_ displays distinct features, particularly in the catalytic region. Unlike other ROs where the active site covering region is unresolved, IPDO_KF1_’s active site is covered by two helices α5 and α6, shielding it from the surface (Fig. 2B). Moreover, helices (α7, α8, and α11) deviate significantly from their counterparts in PDO_KF1_ and RO_CH34_ (Fig. 2B and C), contributing to a restructured active site cavity. These unique features introduce additional residues, such as F249 and I294 (marked in yellow, Fig. 2D), into the active site cavity, which also includes G172, T175, V178, R234, I246, H257, and F333 residues (marked in yellow, Fig. 2D).

### Isophthalate-bound complex crystal structure

**Fig 3:**
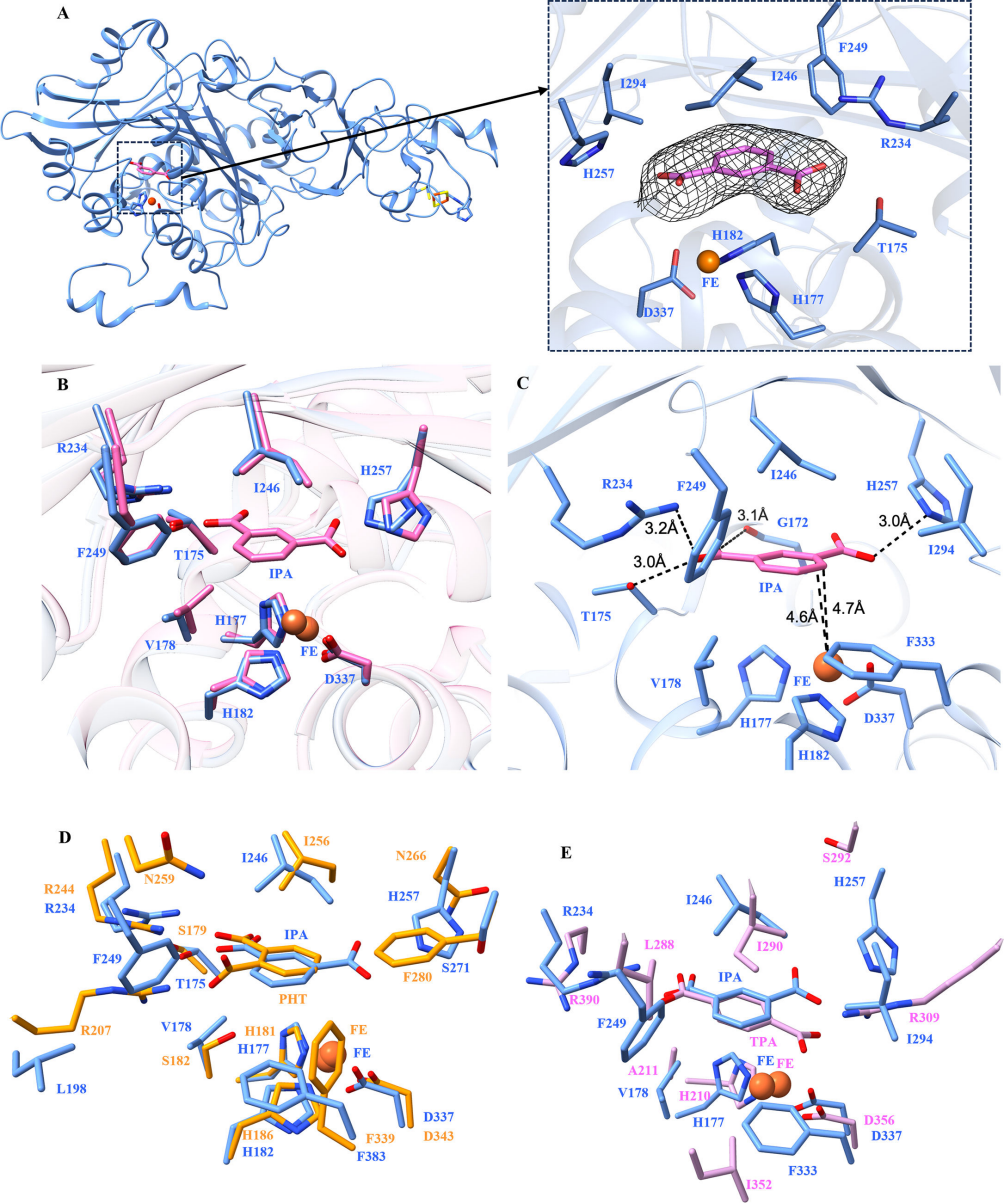
Structure of isophthalate-bound IPDO_KF1_. (**A**) Cartoon representation of an IPDO_KF1_ protomer with bound isophthalate. Left inset: magnified view of the active site showing an electron density polder map (contoured at 3σ) at 3.3 Å resolution for isophthalate (IPA). (**B**) Superposition of active site residues of apo-IPDO_KF1_ and the isophthalate-bound structure (pink) highlights a shift in H257, which moves outward to accommodate the isophthalate molecule. (**C**) The isophthalate-bound IPDO_KF1_ structure reveals crucial active site residues. The distances of isophthalate C3 and C4 carbons from mononuclear iron are shown. The interactions of isophthalate with IPDO_KF1_ active site residues are highlighted. (**D**) The superposition of active site residues of IPDO_KF1_ (blue) with PDO_KF1_ (orange). The substitution of L198 at the corresponding position of R207 of PDO_KF1_ explains IPDO_KF1_’s inability to dihydroxylate phthalate (PHT). (**E**) Superposition of active site residues of IPDO_KF1_ with TPDO_E6_ (pink) indicates that the position of H257 relative to R309 in TPDO_E6_ may account for the inability of IPDO_KF1_ to dihydroxylate terephthalate (TPA).

To identify the residues involved in isophthalate recognition, we co-crystallized IPDO_KF1_ with its substrate and determined the structure of the isophthalate-bound complex to 3.3 Å resolution. Although the resolution was relatively low, the electron density near the mononuclear iron site clearly accommodated the isophthalate molecule, which was unambiguously modeled with a real space correlation coefficient of 0.98 (Fig. 3A). The backbone conformation in the IPDO_KF1_-isophthalate complex structure closely resembles that of the substrate-free IPDO_KF1_ (RMSD 0.585 Å over 419 Cα atoms). Substrate-induced changes are localized primarily to the active site, where H257 shifts by 1.2 Å to accommodate the carboxylate group of isophthalate (Fig. 3B). The isophthalate molecule is oriented in the active site such that its C3 and C4 carbon atoms are located at 4.6 and 4.7 Å distances from the mononuclear iron, respectively (Fig. 3C), consistent with earlier reported distance between mononuclear iron of phthalate dioxygenase and phthalate (3, 44). Interestingly, residues R234 and H257 participate in salt bridge interactions with the C1 and C3 carboxyl groups of isophthalate, positioned at 3.2 and 3.0 Å from R234 and H257, respectively. Additionally, the C1 carboxylate forms hydrogen bonds with the side chain hydroxyl of T175 and the main chain carbonyl of G172 at distances of 3.0 and 3.1 Å, respectively (Fig. 3C). These interactions indicate that the carboxylate groups are key for substrate anchoring within the active site. Furthermore, residues I246 and I294 contribute hydrophobic contacts, while residue F333 engages in π-π stacking interactions with the isophthalate aromatic ring (Fig. 3C).

Next, with the isophthalate-bound complex structure, we investigated the structural basis of IPDO_KF1_’s regiospecificity as a 3,4-dioxygenase. The active site architecture, particularly the positioning of polar residues, restricts isophthalate to a fixed orientation, preventing alternative binding modes. Previously, residue I246 equivalent in PDO_KF1_ (I256) has been shown to be crucial for dioxygenation chemistry, as the I256G variant of PDO_KF1_ produced a mono-oxygenated product in contrast to the di-oxygenated product generated by wild-type PDO_KF1_ (14). Structural alignment suggests that I246 in IPDO_KF1_ likely plays a similar crucial role in facilitating dioxygenation (Fig. 3D). Additionally, nonpolar residues such as F249, I294, and F333 create steric hindrance, prohibiting the substrate from aligning in a manner that would facilitate 2,3- or 4,5-dioxygenation.

Furthermore, we investigated why IPDO_KF1_ lacks activity toward phthalate. We conducted a structural comparison with its homolog PDO_KF1_. Superposition of the two enzymes revealed that while most active site residues are conserved, notable differences arise in the spatial arrangement of residues involved in carboxylate recognition. In PDO_KF1_, residues R207 and R244 that interact with the substrate’s carboxylate groups are located on the same face of the active site. In contrast, IPDO_KF1_ displays a distinct geometry, where R234 and H257, which engage with the carboxylate groups, are situated on opposing sides of the active site pocket (Fig. 3D). Structural alignment further indicates that R234 in IPDO_KF1_ occupies a position equivalent to R244 in PDO_KF1_. However, residues S182 and R207 in PDO_KF1_ (key for phthalate stabilization) are substituted by V178 and L198 in IPDO_KF1_. These changes, particularly the replacement of polar and charged residues with nonpolar residues, likely disrupt essential interactions required for phthalate binding and catalysis, thereby explaining IPDO_KF1_’s inactivity toward phthalate.

Similarly, the structural comparison of the TPDO_E6_ alpha subunit highlighted why IPDO_KF1_ is incapable of catalyzing terephthalate dihydroxylation. While few of the substrate-interacting residues are conserved, such as I246 and R234, the residue equivalent of R309 is lacking in the IPDO_KF1_ structure (Fig. 3E). Instead, H257 is present at the meta-position (in IPDO_KF1_), which explains interaction with the meta-carboxylate group of isophthalate rather than the para-carboxylate group of terephthalate (Fig. 3E). Furthermore, I294 at the equivalent place of R309 of TPDO_E6_ provides steric hindrance to any para-substituted substrates like terephthalate. Overall, this analysis highlights G172, T175, R234, I246, F249, H257, I294, and F333 as key determinants for the specificity of IPDO_KF1_.

### Biochemical activity

**Fig 4:**
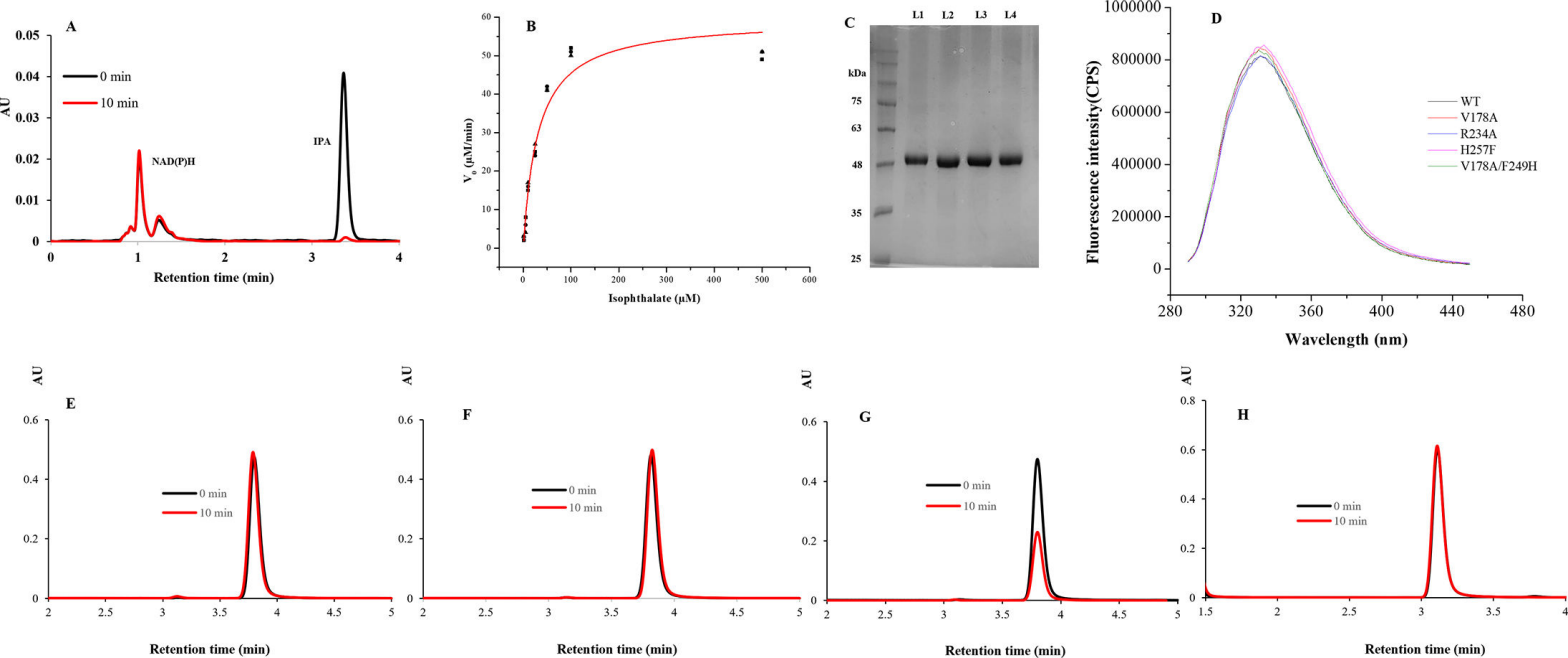
Biochemical and mutagenesis analyses of IPDO_KF1_. (**A**) Conversion of isophthalate (IPA) by IPDO_KF1_ and IPDR_KF1_. Purified enzymes (0.1 µM each) were incubated with 50 µM IPA in the presence of NAD(P)H. (**B**) Dependence of initial velocity of oxygen consumption on the isophthalate concentration in air-saturated buffer. Red lines represent fits of the Michaelis–Menten equation to the data. (**C**) SDS-PAGE gel of IPDO_KF1_ variants purified with Ni-affinity chromatography: (L1) V178A, (L2) R234A, (L3) H257F, and (L4) V178A/F249H. (**D**) Intrinsic fluorescence spectra of wild-type (WT) and variants. The variants exhibited fluorescence profiles comparable to the WT, with similar emission intensity and wavelength maximum (*λ*_max_), indicating preservation of the overall protein fold. Activity reactions of (**E**) R234A variant and (**F**) H257F variant with isophthalate. These variants show complete loss of activity against isophthalate. Reactions of the V178A variant with (**G**) isophthalate and (**H**) phthalate.

For biochemical characterization, IPDO_KF1_ was purified, and iron quantification indicated the presence of 3.1 ± 0.4 iron atoms per monomer. Enzymatic activity toward isophthalate was assessed using a high-performance liquid chromatography (HPLC)-based assay. When equimolar concentrations of IPDO_KF1_ and its reductase partner IPDR_KF1_ were incubated with 50 µM isophthalate in the presence of NAD(P)H (50 mM Tris-HCl, pH 8.0), the substrate was consumed (Fig. 4A). To further analyze the catalytic efficiency, oxygen consumption was monitored under steady-state conditions using an Oxygraph. The assay was performed in air-saturated buffer (50 mM Tris-HCl, pH 8.0 at 25°C), with IPDO_KF1_, IPDR_KF1_, NAD(P)H, and isophthalate. Substrate specificity testing showed that IPDO_KF1_ did not exhibit activity toward several tested compounds (phthalate, terephthalate, 3-chlorobenzoate, and 2-chlorobenzoate), suggesting isophthalate is the preferred substrate (data not shown). The kinetics experiments showed that the initial rate of oxygen consumption obeys Michaelis-Menten kinetics, yielding an apparent *k*_cat_ of 4.96 ± 0.49 s^−1^ and *K*_*M*_ of 30.97 ± 7.28 µM for isophthalate (Table 2; Fig. 4B). A similar *K*_*M*_ value (72 ± 6 µM) was reported for IPDO_E6_ against isophthalate (1). Stoichiometric coupling experiments indicated that the reaction consumes 1.12 ± 0.18 mol of NAD(P)H and 1.2 ± 0.09 mol of molecular oxygen per mole of isophthalate oxidized. Importantly, incubation of the reaction mixture with a dye-decolorizing peroxidase showed no dye decolorization, indicating that no H_2_O_2_ was generated during the catalytic process. These results collectively indicate that IPDO_KF1_ catalyzes isophthalate dihydroxylation through a tightly coupled reaction mechanism involving NAD(P)H and O_2_.

**TABLE 2:** Apparent steady-state kinetic parameters of IPDO_KF1_ and V178A/F249H variant of IPDO_KF1_ for different substrates^*a*^

	Substrate	*K*_*M*_ (µM)	*K*_cat_ (s^−1^)	*K*_cat_/*K*_*M*_ (mM^−1^ s^−1^)	Coupling^*b*^
Wild type	Isophthalate	31 ± 7	4.9 ± 0.5	160 ± 41	1.12 ± 0.18
V178A/F249H	Isophthalate	32 ± 8	4.8 ± 0.3	148 ± 39	1.11 ± 0.21
V178A/F249H	Terephthalate	95 ± 11	4.9 ± 0.2	51 ± 6	1.42 ± 0.45
V178A/F249H	Phthalate	91 ± 16	4.0 ± 0.2	44 ± 8	1.23 ± 0.45

^*a*^ Experiments were performed using air-saturated 50 mM Tris, pH 7.5, at 25°C. The reported parameters are based on oxygen consumption.

^*b*^ Relative amount of NAD(P)H oxidized per mole of aromatic substrate hydroxylated.

### Mutagenesis experiments

To ascribe the role of active site residues, single and double mutants of IPDO_KF1_ were generated. The single variants, V178A, R234A, H257F, and the double variant, V178A/F249H, were purified with equivalent yield (~20 mg per liter of culture) (Fig. 4C), and each retained an iron content ranging from 2.6 to 3.2 ± 0.6 atoms per monomer. Additionally, to assess the structural impact of these mutations, intrinsic fluorescence spectroscopy was performed. The fluorescence emission spectra of all variants closely resembled that of the wild-type (WT) enzyme, displaying similar emission intensities and emission maximum (*λ*_max_) (Fig. 4D), indicating that the overall tertiary structure of the mutants remained largely unaltered.

**Fig 5:**
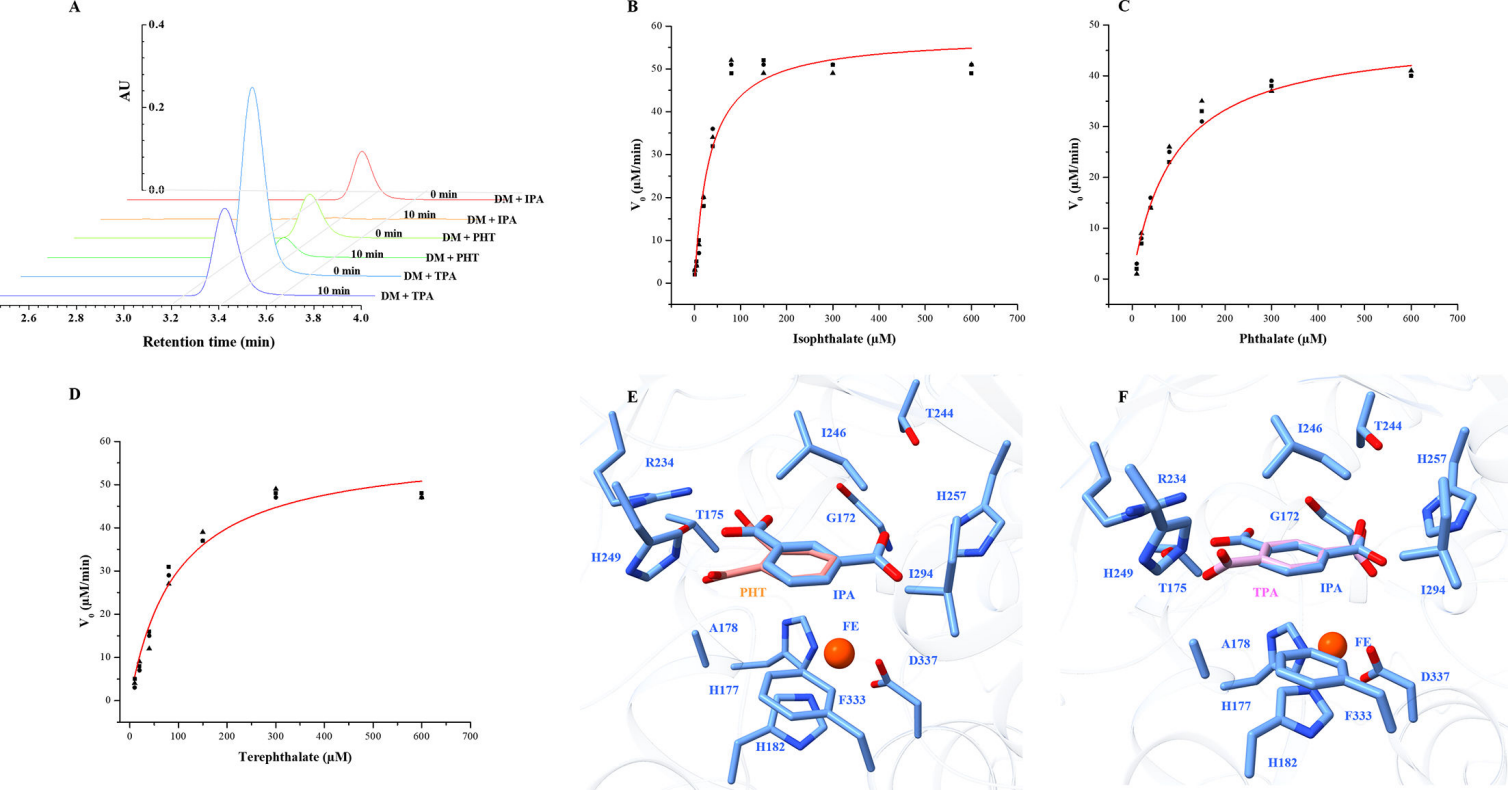
Biochemical and structural analyses of the V178A/F249H IPDO_KF1_ variant. (**A**) Conversion of substrates—isophthalate, IPA; terephthalate, TPA; and phthalate, PHT—by purified double mutant V178A/F249H variant of IPDO_KF1_ (DM). Dependence of initial velocity of oxygen consumption by DM variant on the (**B**) isophthalate concentration, (**C**) phthalate concentration, and (**D**) terephthalate concentration in air-saturated buffer. Red lines represent fits of the Michaelis–Menten equation to the data. Active site of V178A/F249H IPDO_KF1_ variant. (**E**) The PHT-docked structure shows C4 and C5 carbons of the aromatic ring are oriented toward the catalytic iron, positioning them for dihydroxylation and resulting in the formation of phthalate *cis*-4,5-dihydrodiol. (**F**) The TPA-docked structure reveals C1 and C2 carbons are exposed to the iron, favoring dihydroxylation at these positions and suggesting the formation of terephthalate *cis*-1,2-diol.

The substitution of residue R234 and H257—both of which form salt bridges with the isophthalate’s carboxylate group—with Phe and Ala, respectively, resulted in a complete loss of enzymatic activity (Fig. 4E and F). This result highlighted that these residues are crucial to align the isophthalate molecule in its catalytically competent position. To expand the enzyme’s substrate range, we next introduced the V178A single mutation and the V178A/F249H double mutation. Based on the structural comparison with PDO_KF1_, the V178A mutation was designed to enlarge the active site near the ortho face of the aromatic ring, potentially accommodating the ortho-carboxylate group of phthalate. While the V178A variant retained activity against isophthalate, it showed no detectable activity against phthalate (Fig. 4G and H), suggesting that additional interactions on the ortho side of the active site are necessary for stabilizing the substrate, phthalate. To address this, we generated the V178A/F249H double mutant, in which the F249H mutation was intended to introduce potential interactions with the carboxylate group of phthalate. Remarkably, this double mutant variant showed activity against all three regioisomers of phthalate and terephthalate without compromising on the activity against isophthalate (Fig. 5A).

Steady-state kinetic parameters of the double mutant were determined using the Oxygraph assay against phthalate, terephthalate, and isophthalate. The double mutant V178A/F249H showed similar affinity to all these substrates, and the *K*_*M*_ values for isophthalate, phthalate, and terephthalate were 32 ± 8, 91 ± 16, and 95 ± 11 µM, respectively (Table 2; Fig. 5B through D). Interestingly, the reactions with terephthalate, phthalate, and isophthalate were also coupled (Table 2). Despite similar catalytic turnover number (*K*_cat_) across the three substrates, the catalytic efficiency (*K*_cat_/*K*_*M*_) of the double mutant was approximately threefold higher for isophthalate than for terephthalate and phthalate. This enhanced efficiency is primarily attributed to the enzyme’s threefold higher affinity (*K*_*M*_) for isophthalate, reinforcing its native substrate preference.

When compared to other known phthalate-degrading dioxygenases, the engineered IPDO_KF1_ variant displayed lower catalytic efficiency (*K*_cat_/*K*_*M*_). For instance, PDO_KF1_ shows 12-fold higher catalytic efficiency (583 ± 87 mM^−1^ s^−1^) toward phthalate than the V178A/F249H IPDO_KF1_ variant (3). This disparity likely arises from key active site differences, particularly the absence of aromatic ring-stabilizing residues (such as the F280 residue) in IPDO_KF1_ (Fig. 3C). In contrast, the TPDO_KF1_ demonstrates a catalytic efficiency of 57 ± 9 mM^−1^ s^−1^ against terephthalate that is comparable to the engineered variant of IPDO_KF1_ (4). However, terephthalate dihydroxylation by the engineered variant is less efficiently coupled to NAD(P)H oxidation than TPDO_KF1_ (Table 2). The lower coupling may stem from the approximately twofold higher *K*_*M*_ of the engineered IPDO_KF1_ (95 ± 11 µM) variant for terephthalate compared to TPDO_KF1_ (47 ± 6 µM).

Finally, to understand substrate binding orientation and potential hydroxylation sites, molecular docking studies were conducted using phthalate and terephthalate in the active site of the V178A/F249H IPDO_KF1_ variant. For phthalate, the steric hindrance from residues G172 and I294 limits its orientation, positioning the carboxylate groups toward the basic residues R234 and H249 (Fig. 5E). This alignment exposes the C4 and C5 carbons of the aromatic ring to the catalytic iron center, favoring dihydroxylation at these positions and leading to the predicted formation of phthalate *cis*-4,5-dihydrodiol, similar to the product formed by PDO_KF1_. In the case of terephthalate, substrate positioning is constrained by residues I294 and F333, resulting in a new binding mode compared to that observed in TPDO_KF1_ (Fig. 3E). In this orientation, the carboxylate groups are directed toward H249 and H257 (Fig. 5F), aligning the C1 and C2 carbons of terephthalate toward the iron center. This spatial arrangement favors dihydroxylation at these positions, resulting in the formation of terephthalate *cis*-1,2-diol product, consistent with the product reported for TPDO_KF1_.

## DISCUSSION

Enzyme engineering plays a pivotal role in developing improved biocatalysts for applications in biotechnology, chemical synthesis, pharmaceuticals, and environmental cleanup (45). Traditionally, rational protein design has focused on modifying residues within the active site to impart desirable catalytic traits. Nonetheless, a major hurdle in achieving improved or novel enzymatic functions is the complexity of understanding how structural features dictate substrate recognition and reaction specificity (14). Our recent work has established the structure-function relationships of two phthalate-oxygenating enzymes, PDO and TPDO, respectively (3, 4). Building on this, we now elucidate the crystal structure of IPDO_KF1_ that initiates the catabolism of isophthalate. By resolving the complex crystal structure of IPDO_KF1_ with isophthalate, we identify the critical features that govern substrate selectivity. Furthermore, leveraging these insights, we engineered an IPDO_KF1_ variant capable of catalyzing all three phthalate regioisomers: isophthalate, phthalate, and terephthalate.

The crystal structure of IPDO_KF1_ reveals the unique architecture of this enzyme compared to TPDO_KF1_ and PDO_KF1_. Notably, while the TPDO_KF1_ and PDO_KF1_ are heterohexameric (α_3_β_3_) and homohexameric (α_3_α_3_), respectively, the IPDO_KF1_ is a homotrimeric (α_3_). Unlike its homologs, PDO_KF1_ and RO_CH34_ (3), it features a structured active site covering the region formed by two helices, α9 and α10. The corresponding region is disordered in other distantly related ROs as well, such as biphenyl dioxygenase from *Burkholderia xenovorans* LB400 (BPDO_LB400_) (29), terephthalate dioxygenase from *Comamonas testosteroni* KF1 (TPDO_KF1_) (4, 34), and salicylate 5-hydroxylase from *Ralstonia* sp. U2 (NagGH) (46). Additionally, structural changes in IPDO_KF1_ compared to other ROs introduce unique active site residues, such as F249 and I294, which contribute to its selective binding of isophthalate.

The isophthalate-bound complex structure validates dihydroxylation at the C3 and C4 positions, consistent with previous ESI-MS analysis that identified the product as isophthalate-3,4-*cis*-diol (13). The structure reveals that the C3 and C4 carbons are positioned closest to the catalytic mononuclear iron center at distances of 4.6 and 4.7 Å, respectively, reinforcing their roles as the primary iron-attacking sites. Additionally, studies on PDO have demonstrated that I256 is critical for orienting the substrate’s aromatic ring to position two carbon atoms near the catalytic iron center for dioxygenation (14), as the I256G variant yielded a monooxygenated product. Based on the structural alignment, I246 in IPDO_KF1_ (corresponding to I256 of PDO) can be predicted to serve a similar role in aligning the C3 and C4 of isophthalate in a plane that facilitates iron-mediated side-on O_2_ binding, a mechanism previously proposed (24, 47).

The crystal structure of the isophthalate-bound IPDO_KF1_ complex, combined with functional studies of key variants, highlights the essential roles of R234 and H257 in conferring substrate specificity. These two residues, positioned on opposing sides of the active site, form salt bridge interactions with the isophthalate carboxyl groups. In agreement with previous findings (1), IPDO_KF1_ displayed no detectable catalytic activity toward phthalate or terephthalate. Structural insights reveal that residues such as V178, F249, and I294 within the active site likely impose steric constraints at the ortho- and para-positions, hindering the accommodation of phthalate or terephthalate. Additionally, the absence of critical carboxylate-interacting residues typically found in other dioxygenases—like R207 in PDO_KF1_ (ortho-face) and R309 in TPDO_KF1_ (para-face)—further restricts IPDO_KF1_’s ability to process these alternative substrates.

Mutagenesis of IPDO_KF1_ introduces additional functionality, enabling the dihydroxylation of phthalate and terephthalate without diminishing isophthalate dihydroxylation activity. The double mutant V178A/F249H was engineered to accommodate the ortho- and para-substituted phthalate and terephthalate molecules. The substitution of V178 to Ala creates space to accommodate the carboxylate group, while the substitution of F249H introduced a new carboxylate-interacting side chain at the ortho face of the active site, mimicking essential interactions observed in PDO_KF1_ and TPDO_KF1_. This engineered variant successfully catalyzed the dihydroxylation of all three phthalate isomers.

Kinetics analysis showed that while the catalytic efficiency of the engineered variant for phthalate was lower than that of specialized wild-type enzymes, the activity remained within a comparable range for terephthalate. The reduced catalytic efficiency toward phthalate may be attributed to the absence of an aromatic ring-stabilizing residue, such as residue F280 in PDO_KF1_, which is not conserved in IPDO_KF1_. Structural modeling and molecular docking illuminated how specific residues, such as G172, I294, and F333, shape the active site landscape to direct the regioselectivity of hydroxylation. The altered orientation of carboxylate groups in the engineered variant guided hydroxylation at the C4 and C5 carbons for phthalate and C1 and C2 carbons for terephthalate, resulting in the formation of phthalate *cis*-4,5-dihydrodiol and terephthalate *cis*-1,2-diol, respectively, similar to the product formed by PDO_KF1_ and TPDO_KF1_.

In summary, this work presents the first structural characterization of IPDO. Through a combination of structural and biochemical approaches, we uncover the molecular underpinnings of IPDO_KF1_’s ability to selectively dihydroxylate isophthalate, revealing key determinants of both substrate specificity and regiospecificity. Targeted mutagenesis further highlights the potential for tuning active site residues to broaden the enzyme’s activity, allowing efficient conversion of all phthalate regioisomers. Collectively, these findings provide a framework for the rational design of RO enzymes with tailored catalytic profiles for biotechnological and environmental applications.

## MATERIALS AND METHODS

### Chemicals, reagents, and bacterial strains

All reagents were of analytical grade. Enzymes used for cloning were from New England Biolabs. Water for buffers was purified using a Merck Synergy Water Purification System to a resistance of at least 18.2 MΩ. *Comamonas testosteroni* KF1 was purchased from the German Collection of Microorganisms and Cell Cultures, DSMZ (Germany).

### DNA manipulation

DNA was purified, amplified, propagated, and cloned using standard protocols (48). Genomic DNA from *Comamonas testosteroni* KF1 was used as the template to amplify genes encoding IPDO_KF1_ components. PCR was carried out using gene-specific primers listed in Table 3. Both the PCR products and the corresponding expression vectors were digested with the appropriate restriction enzymes, purified, and ligated using T4 DNA ligase. The resulting ligation mixture was initially transformed into competent cells. Verified recombinant plasmids were then introduced into *E. coli* BL21 (DE3) cells for expression. For each construct, multiple individual colonies were selected and cultured overnight at 37°C in 10 mL LB medium containing kanamycin (50 µg/mL) with shaking at 220 rpm. Plasmid DNA was isolated and screened via restriction digestion and DNA sequencing. Site-directed mutagenesis was performed using overlapping PCR with mutagenic primers (Table 3) to introduce desired amino acid substitutions. Each 25 µL reaction contained 1× Phusion buffer (HF), 100 µM dNTPs, 2 ng/µL plasmid template, 1 µM of each primer, 1 U of Phusion DNA polymerase, and 1% DMSO. PCR amplification success was confirmed by agarose gel electrophoresis. The parental template was digested with DpnI at 37°C for 1 hour, and 5 µL of the reaction was transformed into chemically competent *E. coli* DH5α cells. Mutant constructs were validated by DNA sequencing.

**TABLE 3:** Primers for cloning genes and mutagenesis used in this study

Name	Sequence
Cloning	
IPDO-FP	5′-AAGCGGGCTAGCATGAACAAGGAAATGTCCGAG-3′
IPDO-RP	5′-ATCGGGAAGCTTTCAGAGGCTGAAGACTGG-3′
IPDR-FP	5′-AAGCGGCATATGGCCTCTACATATCTTCAG-3′
IPDR-RP	5′-AAGAGGCTCGAGTCAGCAATCCAGGACCAG-3′
Mutagenesis	
IPDOV178A-FP	5′-GACACTGCCCATGCGTCATATGTGCAC-3′
IPDOV178A-RP	5′-GTGCACATATGACGCATGGGCAGTGTC-3′
IPDOR234A-FP	5′-CTCTTACTACTGGGCCATCACGCAGTGGC-3′
IPDOR234A-RP	5′-GCCACTGCGTGATGGCCCAGTAGTAAGAG-3′
IPDOF249H-FP	5′-GCTCATTGCACCGCACGGCAACCATGC-3′
IPDOF249H-RP	5′-GCATGGTTGCCGTGCGGTGCAATGAGC-3′
IPDOH257F-FP	5′-CATGCACTGGGCGGTTTTGTGTGGGTGCCTATC-3′
IPDOH257F-RP	5′-GATAGGCACCCACACAAAACCGCCCAGTGCATG-3′

### Expression and purification

*E. coli* BL21 (DE3) cells were cultivated in LB medium containing kanamycin (50 µg/mL) at 37°C with shaking at 200 rpm until the optical density at 600 nm reached ~0.6. At this point, cultures were shifted to 16°C, and protein expression was induced with 0.5 mM isopropyl β-D-thiogalactopyranoside for 16 hours. Cells were harvested by centrifugation (6,000 × *g*, 10 min, and 4°C) and stored at −80°C until further use. For protein purification, cell pellets were resuspended in 10 mL of lysis buffer (20 mM Tris, pH 7.4, and 300 mM NaCl) supplemented with 5 mM PMSF. Cells were lysed using a Constant Cell Disruptor operated at 20 psi. Lysates were clarified by centrifugation, and the resulting supernatants were loaded onto Ni-NTA affinity columns pre-equilibrated with lysis buffer. Target proteins were eluted using a linear imidazole gradient ranging from 100 to 500 mM in the same buffer. Eluted fractions were analyzed on SDS-PAGE gel, and proteins were found to be >95% pure, with typical yields of ∼20 mg of purified protein per liter of culture. Purified proteins were concentrated using an Amicon Millipore filter with a 10 kDa molecular weight cutoff and dialyzed with 25 mM HEPES, pH 7.4, and 150 mM NaCl. Size exclusion chromatography analysis revealed that the IPDO_KF1_ exists as a ~150 kDa species, consistent with a homotrimeric (α_3_) assembly.

### Analytical methods

Protein concentration was estimated by measuring absorbance at 280 nm (*A*_280_) using a Cary 300 UV-visible spectrophotometer (49). Iron content was quantified using Ferene-S calorimetric assay (50), with ferrous ammonium sulfate serving as the standard. For iron estimation, various concentrations of protein were diluted to 0.4 mL with double-distilled water, followed by the addition of 0.05 mL of 8 N HCl. After a 10-min incubation, 0.05 mL of 80% trichloroacetic acid was added to precipitate the protein, which was then removed by centrifugation. The supernatant (100 µL) was mixed with 900 µL of a working Ferene-S solution (5 mM Ferene-S and 0.2 M L-ascorbic acid in 0.4 M ammonium acetate buffer, pH 4.3). The absorbance at 595 nm was recorded after 30 min at room temperature to determine the iron concentration (50, 51). To assess structural changes induced by mutation, fluorescence spectroscopy was employed, utilizing the intrinsic fluorescence of aromatic amino acids. Fluorescence measurements were performed using a Fluoromax plus Spectrofluorometer (Horiba Jobin Yvon), with an excitation wavelength of 280 nm and emission spectra collected in the range of 290–450 nm using excitation and emission slits set at 3 nm each.

### Crystallization

Crystallization trials of IPDO_KF1_ were performed using the sitting-drop vapor diffusion method in 96-well plates (Hampton Research) at 293 K. Equal volumes (1:1) of protein solution and reservoir solution were mixed and equilibrated against 50 µL of reservoir solution (52). Crystals suitable for diffraction were obtained under optimized conditions containing 0.4 M potassium thiocyanate, 0.1 M Tris-HCl (pH 7.0), and 15% (wt/vol) polyethylene glycol (PEG) 3350. To obtain the isophthalate-bound complex, IPDO_KF1_ was co-crystallized in the presence of 5 mM isophthalate. Crystals were grown in a condition containing 0.1 M Tris-HCl (pH 7.0), 0.2 M ammonium fluoride, and 15% (wt/vol) PEG 3350. Prior to data collection, crystals were cryoprotected by briefly soaking in reservoir solution supplemented with 20% (vol/vol) ethylene glycol and flash-cooled in a nitrogen stream at 100 K.

### X-ray data collection and structure determination

X-ray diffraction data were collected at the European Synchrotron Radiation Facility, Grenoble, France, and Home Source, Macromolecular Crystallography Unit, IIC, IIT Roorkee. The data quality was evaluated using Aimless (53) from the CCP4i suite (54). Initial phase determination was accomplished via molecular replacement using MOLREP (CCP4 Version) (55), with the structure of the AlphaFold model of IPDO_KF1_ serving as the search model. The model building was performed using Coot (56), and the structure refinement was performed using REFMAC5 (57). Solvent molecules were added at positions where the Fo−Fc difference electron density exceeded 3σ and where the 2Fo−2Fc maps displayed corresponding density at the 1σ level. Iterative cycles of rigid-body refinement followed by restrained refinement were employed to optimize geometry and reduce *R*_cryst_ and *R*_free_ values to acceptable levels. A summary of data collection and refinement statistics is provided in Table 1. All structural figures of protein and ligand structures were generated using PyMol (58) and UCSF Chimera (59).

### Modeling and molecular docking

The structural model of CbaDO and mutants of IPDO_KF1_ was generated using AlphaFold (41). To investigate ligand binding, flexible molecular docking was conducted using AutoDock Vina (60). Prior to docking, a dioxygen molecule was modeled in a side-on orientation at the mononuclear iron center. The distances from the dioxygen atoms to the iron were set at 2.2 and 2.3 Å based on the coordination geometry described previously (24). Ligand structures were drawn using ChemSketch (61) and subsequently prepared for docking by merging non-polar hydrogen atoms onto both the ligands and the protein models using AutoDock Tools. For receptor preparation in CbaDO, residues R235, E175, and R259 were selected to allow side-chain flexibility. Similarly, for IPDO_KF1_ mutants, residues R234, H249, and H257 were selected to allow side-chain flexibility. The docking grid was generated using AutoGrid4, with the box dimensions set to 40 × 40 × 40 Å for all the receptors. Ten docking conformations were generated for each enzyme-ligand pair, and the most favorable binding poses were selected based on spatial orientation and interaction energy.

### Biochemical activity

The enzymatic activity of IPDO_KF1_ was assessed by quantifying isophthalate depletion using HPLC. HPLC analyses were conducted on an X-Bridge (Waters, Milford, USA) C18 column (5 µm, 150 × 4.6 mm) connected to a Waters HPLC system (Milford, USA) equipped with a binary pump (Waters; Binary pump 1525) and a photodiode array detector (Waters; PDA 2998). Chromatographic data were acquired and processed using EMPOWER software (version 3, feature release 4). Reactions were carried out in 1 mL assay mixtures containing 50 mM Tris-HCl (pH 8.0), 100 µM Fe(NH_4_)_2_(SO_4_)_2_·6H_2_O, 50 µM isophthalate, 200 µM NAD(P)H, and 0.25 µM of both IPDO_KF1_ and IPDR_KF1_. Aliquots (100 µL) were withdrawn at specific time points and quenched with an equal volume of acetonitrile (100%) before HPLC analysis. The mobile phase comprised 68% acidified water and 32% acetonitrile, with a flow rate of 1 mL/min. Detection was carried out at 240 nm. For substrate specificity analysis, IPDO_KF1_ was incubated with 50 µM of various aromatic compounds, including phthalate, isophthalate, terephthalate, 3-chlorobenzoate, 2-chlorobenzoate, 3-phenoxybenzoate, or 4-phenoxybenzoate under the same reaction conditions, and the products were analyzed via HPLC. For the activity of double mutant V178A/F249H, 200 µM of phthalate, isophthalate, and terephthalate was used.

Steady-state kinetic parameters were determined by monitoring O_2_ consumption using a Clark-type polarographic oxygen electrode (OXYG1; Hansatech Instruments) connected to a circulating water bath. Assays were performed in 1 mL of air-saturated 50 mM Tris-HCl (pH 8.0) at 25°C, containing 100 µM Fe(NH_4_)_2_(SO_4_)_2_·6H_2_O, 1 mM NAD(P)H, and 0.25 µM each of IPDO_KF1_ and IPDR_KF1_. Reactions were initiated by substrate addition. The electrode was calibrated with air-saturated water and O_2_-depleted water (prepared using the addition of sodium dithionite). Reaction rates were corrected for the baseline oxygen consumption, measured prior to the aromatic substrate addition. Initial velocities were used to calculate steady-state kinetic parameters by fitting data to the Michaelis–Menten equation.

## Data Availability

The IPDO_KF1_ and IPDO_KF1_ complex with isophthalate coordinates and structure factors have been deposited in the Protein Data Bank with accession numbers 9INA and 9IOY. Other data are available from the corresponding author upon reasonable request.

## References

[1] Fukuhara Y, Inakazu K, Kodama N, Kamimura N, Kasai D, Katayama Y, Fukuda M, Masai E. 2010. Characterization of the isophthalate degradation genes of Comamonas sp. strain E6. Appl Environ Microbiol 76:519–527. doi:10.1128/AEM.01270-09 19933340 PMC2805221

[2] Lin H, Ge R-S, Chen G-R, Hu G-X, Dong L, Lian Q-Q, Hardy DO, Sottas CM, Li X-K, Hardy MP. 2008. Involvement of testicular growth factors in fetal Leydig cell aggregation after exposure to phthalate in utero. Proc Natl Acad Sci USA 105:7218–7222. doi:10.1073/pnas.0709260105 18469139 PMC2438230

[3] Mahto JK, Neetu N, Waghmode B, Kuatsjah E, Sharma M, Sircar D, Sharma AK, Tomar S, Eltis LD, Kumar P. 2021. Molecular insights into substrate recognition and catalysis by phthalate dioxygenase from Comamonas testosteroni. J Biol Chem 297:101416. doi:10.1016/j.jbc.2021.101416 34800435 PMC8649396

[4] Mahto JK, Neetu N, Sharma M, Dubey M, Vellanki BP, Kumar P. 2022. Structural insights into dihydroxylation of terephthalate, a product of polyethylene terephthalate degradation. J Bacteriol 204:e0054321. doi:10.1128/JB.00543-21 35007143 PMC8923216

[5] Benjamin S, Masai E, Kamimura N, Takahashi K, Anderson RC, Faisal PA. 2017. Phthalates impact human health: epidemiological evidences and plausible mechanism of action. J Hazard Mater 340:360–383. doi:10.1016/j.jhazmat.2017.06.036 28800814

[6] Benjamin S, Pradeep S, Josh MS, Kumar S, Masai E. 2015. A monograph on the remediation of hazardous phthalates. J Hazard Mater 298:58–72. doi:10.1016/j.jhazmat.2015.05.004 26004054

[7] Xie M, Wu Y, Little JC, Marr LC. 2016. Phthalates and alternative plasticizers and potential for contact exposure from children’s backpacks and toys. J Expo Sci Environ Epidemiol 26:119–124. doi:10.1038/jes.2015.71 26531804

[8] Verma S, Choudhary S, Amith Kumar K, Mahto JK, Vamsi K AK, Mishra I, Prakash VB, Sircar D, Tomar S, Kumar Sharma A, Singla J, Kumar P. 2025. Mechanistic and structural insights into EstS1 esterase: a potent broad-spectrum phthalate diester degrading enzyme. Structure 33:247–261. doi:10.1016/j.str.2024.11.006 39642872

[9] Engel SM, Patisaul HB, Brody C, Hauser R, Zota AR, Bennet DH, Swanson M, Whyatt RM. 2021. Neurotoxicity of ortho-phthalates: recommendations for critical policy reforms to protect brain development in children. Am J Public Health 111:687–695. doi:10.2105/AJPH.2020.306014 33600256 PMC7958063

[10] López-Carrillo L, Hernández-Ramírez RU, Calafat AM, Torres-Sánchez L, Galván-Portillo M, Needham LL, Ruiz-Ramos R, Cebrián ME. 2010. Exposure to phthalates and breast cancer risk in Northern Mexico. Environ Health Perspect 118:539–544. doi:10.1289/ehp.0901091 20368132 PMC2854732

[11] Moore RW, Rudy TA, Lin T-M, Ko K, Peterson RE. 2001. Abnormalities of sexual development in male rats with in utero and lactational exposure to the antiandrogenic plasticizer Di(2-ethylhexyl) phthalate. Environ Health Perspect 109:229–237. doi:10.1289/ehp.01109229 11333183 PMC1240240

[12] Radke EG, Braun JM, Meeker JD, Cooper GS. 2018. Phthalate exposure and male reproductive outcomes: a systematic review of the human epidemiological evidence. Environ Int 121:764–793. doi:10.1016/j.envint.2018.07.029 30336412 PMC10825890

[13] Wang YZ, Zhou Y, Zylstra GJ. 1995. Molecular analysis of isophthalate and terephthalate degradation by Comamonas testosteroni YZW-D. Environ Health Perspect 103:9–12. doi:10.1289/ehp.95103s49 PMC1519302 8565920

[14] Tian J, Liu J, Knapp M, Donnan PH, Boggs DG, Bridwell-Rabb J. 2023. Custom tuning of rieske oxygenase reactivity. Nat Commun 14:5858. doi:10.1038/s41467-023-41428-x 37730711 PMC10511449

[15] Dumitru R, Jiang WZ, Weeks DP, Wilson MA. 2009. Crystal structure of dicamba monooxygenase: a rieske nonheme oxygenase that catalyzes oxidative demethylation. J Mol Biol 392:498–510. doi:10.1016/j.jmb.2009.07.021 19616011 PMC3109874

[16] Robert LD, Rydel TJ, Storek MJ, Sturman EJ, Moshiri F, Bartlett RK, Brown GR, Eilers RJ, Dart C, Qi Y. 2009. Dicamba monooxygenase: structural insights into a dynamic rieske oxygenase that catalyzes an exocyclic monooxygenation. J Mol Biol 392:481–497. doi:10.1016/j.jmb.2009.07.022 19616009

[17] Inoue K, Ashikawa Y, Umeda T, Abo M, Katsuki J, Usami Y, Noguchi H, Fujimoto Z, Terada T, Yamane H, Nojiri H. 2009. Specific interactions between the ferredoxin and terminal oxygenase components of a class IIB rieske nonheme iron oxygenase, carbazole 1,9a-dioxygenase. J Mol Biol 392:436–451. doi:10.1016/j.jmb.2009.07.029 19616558

[18] Carredano E, Karlsson A, Kauppi B, Choudhury D, Parales RE, Parales JV, Lee K, Gibson DT, Eklund H, Ramaswamy S. 2000. Substrate binding site of naphthalene 1,2-dioxygenase: functional implications of indole binding. J Mol Biol 296:701–712. doi:10.1006/jmbi.1999.3462 10669618

[19] Furusawa Y, Nagarajan V, Tanokura M, Masai E, Fukuda M, Senda T. 2004. Crystal structure of the terminal oxygenase component of biphenyl dioxygenase derived from Rhodococcus sp. strain RHA1. J Mol Biol 342:1041–1052. doi:10.1016/j.jmb.2004.07.062 15342255

[20] Nojiri H, Ashikawa Y, Noguchi H, Nam J-W, Urata M, Fujimoto Z, Uchimura H, Terada T, Nakamura S, Shimizu K, Yoshida T, Habe H, Omori T. 2005. Structure of the terminal oxygenase component of angular dioxygenase, carbazole 1,9a-dioxygenase. J Mol Biol 351:355–370. doi:10.1016/j.jmb.2005.05.059 16005887

[21] Kim JH, Kim BH, Brooks S, Kang SY, Summers RM, Song HK. 2019. Structural and mechanistic insights into caffeine degradation by the bacterial N-demethylase complex. J Mol Biol 431:3647–3661. doi:10.1016/j.jmb.2019.08.004 31412262

[22] Parales RE, Parales JV, Gibson DT. 1999. Aspartate 205 in the catalytic domain of naphthalene dioxygenase is essential for activity. J Bacteriol 181:1831–1837. doi:10.1128/JB.181.6.1831-1837.1999 10074076 PMC93582

[23] Kauppi B, Lee K, Carredano E, Parales RE, Gibson DT, Eklund H, Ramaswamy S. 1998. Structure of an aromatic-ring-hydroxylating dioxygenase-naphthalene 1,2-dioxygenase. Structure 6:571–586. doi:10.1016/s0969-2126(98)00059-8 9634695

[24] Karlsson A, Parales JV, Parales RE, Gibson DT, Eklund H, Ramaswamy S. 2003. Crystal structure of naphthalene dioxygenase: side-on binding of dioxygen to iron. Science 299:1039–1042. doi:10.1126/science.1078020 12586937

[25] Batie CJ, LaHaie E, Ballou DP. 1987. Purification and characterization of phthalate oxygenase and phthalate oxygenase reductase from Pseudomonas cepacia. J Biol Chem 262:1510–1518. doi:10.1016/S0021-9258(19)75664-6 3805038

[26] Bertini I, Luchinat C, Mincione G, Parigi G, Gassner GT, Ballou DP. 1996. NMRD studies on phthalate dioxygenase: evidence for displacement of water on binding substrate. J Biol Inorg Chem 1:468–475. doi:10.1007/s007750050080

[27] Tarasev M, Rhames F, Ballou DP. 2004. Rates of the phthalate dioxygenase reaction with oxygen are dramatically increased by interactions with phthalate and phthalate oxygenase reductase. Biochemistry 43:12799–12808. doi:10.1021/bi0490587 15461452

[28] Tarasev M, Ballou DP. 2005. Chemistry of the catalytic conversion of phthalate into its cis-dihydrodiol during the reaction of oxygen with the reduced form of phthalate dioxygenase. Biochemistry 44:6197–6207. doi:10.1021/bi047724y 15835907

[29] Kumar P, Mohammadi M, Viger J-F, Barriault D, Gomez-Gil L, Eltis LD, Bolin JT, Sylvestre M. 2011. Structural insight into the expanded PCB-degrading abilities of a biphenyl dioxygenase obtained by directed evolution. J Mol Biol 405:531–547. doi:10.1016/j.jmb.2010.11.009 21073881 PMC3102011

[30] Kumar P, Mohammadi M, Dhindwal S, Pham TTM, Bolin JT, Sylvestre M. 2012. Structural insights into the metabolism of 2-chlorodibenzofuran by an evolved biphenyl dioxygenase. Biochem Biophys Res Commun 421:757–762. doi:10.1016/j.bbrc.2012.04.078 22546558

[31] Colbert CL, Agar NYR, Kumar P, Chakko MN, Sinha SC, Powlowski JB, Eltis LD, Bolin JT. 2013. Structural characterization of Pandoraea pnomenusa B-356 biphenyl dioxygenase reveals features of potent polychlorinated biphenyl-degrading enzymes. PLoS One 8:e52550. doi:10.1371/journal.pone.0052550 23308114 PMC3536784

[32] Liu J, Tian J, Perry C, Lukowski AL, Doukov TI, Narayan ARH, Bridwell-Rabb J. 2022. Design principles for site-selective hydroxylation by a rieske oxygenase. Nat Commun 13:1–13. doi:10.1038/s41467-021-27822-3 35017498 PMC8752792

[33] Lukowski AL, Liu J, Bridwell-Rabb J, Narayan ARH. 2020. Structural basis for divergent C-H hydroxylation selectivity in two rieske oxygenases. Nat Commun 11:2991. doi:10.1038/s41467-020-16729-0 32532989 PMC7293229

[34] Kincannon WM, Zahn M, Clare R, Lusty Beech J, Romberg A, Larson J, Bothner B, Beckham GT, McGeehan JE, DuBois JL. 2022. Biochemical and structural characterization of an aromatic ring–hydroxylating dioxygenase for terephthalic acid catabolism. Proc Natl Acad Sci USA 119. doi:10.1073/pnas.2121426119 PMC9060491 35312352

[35] Wissner JL, Escobedo-Hinojosa W, Vogel A, Hauer B. 2021. An engineered toluene dioxygenase for a single step biocatalytical production of (-)-(1S,2R)-cis-1,2-dihydro-1,2-naphthalenediol. J Biotechnol 326:37–39. doi:10.1016/j.jbiotec.2020.12.007 33359214

[36] Wissner JL, Schelle JT, Escobedo‐Hinojosa W, Vogel A, Hauer B. 2021. Semi‐rational engineering of toluene dioxygenase from Pseudomonas putida F1 towards oxyfunctionalization of bicyclic aromatics. Adv Synth Catal 363:4905–4914. doi:10.1002/adsc.202100296

[37] Parales RE, Lee K, Resnick SM, Jiang H, Lessner DJ, Gibson DT. 2000. Substrate specificity of naphthalene dioxygenase: effect of specific amino acids at the active site of the enzyme. J Bacteriol 182:1641–1649. doi:10.1128/JB.182.6.1641-1649.2000 10692370 PMC94462

[38] Yu CL, Parales RE, Gibson DT. 2001. Multiple mutations at the active site of naphthalene dioxygenase affect regioselectivity and enantioselectivity. J Ind Microbiol Biotechnol 27:94–103. doi:10.1038/sj.jim.7000168 11641767

[39] Halder JM, Nestl BM, Hauer B. 2018. Semirational engineering of the naphthalene dioxygenase from Pseudomonas sp. NCIB 9816-4 towards selective asymmetric dihydroxylation. ChemCatChem 10:178–182. doi:10.1002/cctc.201701262

[40] Dhindwal S, Gomez-Gil L, Neau DB, Pham TTM, Sylvestre M, Eltis LD, Bolin JT, Kumar P. 2016. Structural basis of the enhanced pollutant-degrading capabilities of an engineered biphenyl dioxygenase. J Bacteriol 198:1499–1512. doi:10.1128/JB.00952-15 26953337 PMC4859605

[41] Jumper J, Evans R, Pritzel A, Green T, Figurnov M, Ronneberger O, Tunyasuvunakool K, Bates R, Žídek A, Potapenko A, et al.. 2021. Highly accurate protein structure prediction with AlphaFold. Nature 596:583–589. doi:10.1038/s41586-021-03819-2 34265844 PMC8371605

[42] Pinto A, Tarasev M, Ballou DP. 2006. Substitutions of the “bridging” aspartate 178 result in profound changes in the reactivity of the rieske center of phthalate dioxygenase. Biochemistry 45:9032–9041. doi:10.1021/bi060216z 16866348

[43] Nakatsu CH, Wyndham RC. 1993. Cloning and expression of the transposable chlorobenzoate-3,4-dioxygenase genes of Alcaligenes sp. strain BR60. Appl Environ Microbiol 59:3625–3633. doi:10.1128/aem.59.11.3625-3633.1993 8285670 PMC182508

[44] Tierney DL, Gassner GT, Luchinat C, Bertini I, Ballou DP, Penner-Hahn JE. 1999. NMR characterization of substrate binding in the phthalate dioxygenase system. Biochemistry 38:11051–11061. doi:10.1021/bi990431y 10460160

[45] Brimberry M, Garcia AA, Liu J, Tian J, Bridwell-Rabb J. 2023. Engineering Rieske oxygenase activity one piece at a time. Curr Opin Chem Biol 72:102227. doi:10.1016/j.cbpa.2022.102227 36410250 PMC9939785

[46] Hou Y-J, Guo Y, Li D-F, Zhou N-Y. 2021. Structural and biochemical analysis reveals a distinct catalytic site of salicylate 5-monooxygenase NagGH from rieske dioxygenases. Appl Environ Microbiol 87:e01629-20. doi:10.1128/AEM.01629-20 33452034 PMC8105011

[47] Kovaleva EG, Lipscomb JD. 2008. Versatility of biological non-heme Fe(II) centers in oxygen activation reactions. Nat Chem Biol 4:186–193. doi:10.1038/nchembio.71 18277980 PMC2720164

[48] Sambrook J, Fritsch EF, Maniatis T. 1989. Molecular cloning: a laboratory manual. Cold spring harbor laboratory press.

[49] Gill SC, von Hippel PH. 1989. Calculation of protein extinction coefficients from amino acid sequence data. Anal Biochem 182:319–326. doi:10.1016/0003-2697(89)90602-7 2610349

[50] Hedayati M, Abubaker-Sharif B, Khattab M, Razavi A, Mohammed I, Nejad A, Wabler M, Zhou H, Mihalic J, Gruettner C, DeWeese T, Ivkov R. 2018. An optimised spectrophotometric assay for convenient and accurate quantitation of intracellular iron from iron oxide nanoparticles. Int J Hyperthermia 34:373–381. doi:10.1080/02656736.2017.1354403 28758530 PMC5871594

[51] Zabinski R, Münck E, Champion PM, Wood JM. 1972. Kinetic and mössbauer studies on the mechanism of protocatechuic acid 4,5-oxygenase. Biochemistry 11:3212–3219. doi:10.1021/bi00767a012 5048285

[52] Mahto JK, Kayastha A, Kumar P. 2024. Expression, purification, kinetics, and crystallization of non-heme mononuclear iron enzymes: biphenyl, phthalate, and terephthalate dioxygenases. Meth Enzymol 704:39–58. doi:10.1016/bs.mie.2024.05.014 39300656

[53] Evans PR, Murshudov GN. 2013. How good are my data and what is the resolution? Acta Crystallogr D Biol Crystallogr 69:1204–1214. doi:10.1107/S0907444913000061 23793146 PMC3689523

[54] Winn MD, Ballard CC, Cowtan KD, Dodson EJ, Emsley P, Evans PR, Keegan RM, Krissinel EB, Leslie AGW, McCoy A, McNicholas SJ, Murshudov GN, Pannu NS, Potterton EA, Powell HR, Read RJ, Vagin A, Wilson KS. 2011. Overview of the CCP4 suite and current developments. Acta Crystallogr D Biol Crystallogr 67:235–242. doi:10.1107/S0907444910045749 21460441 PMC3069738

[55] Vagin A, Teplyakov A. 2010. Molecular replacement with MOLREP. Acta Crystallogr D Biol Crystallogr 66:22–25. doi:10.1107/S0907444909042589 20057045

[56] Emsley P, Cowtan K. 2004. Coot: model-building tools for molecular graphics. Acta Crystallogr D Biol Crystallogr 60:2126–2132. doi:10.1107/S0907444904019158 15572765

[57] Murshudov GN, Skubák P, Lebedev AA, Pannu NS, Steiner RA, Nicholls RA, Winn MD, Long F, Vagin AA. 2011. REFMAC5 for the refinement of macromolecular crystal structures. Acta Crystallogr D Biol Crystallogr 67:355–367. doi:10.1107/S0907444911001314 21460454 PMC3069751

[58] DeLano WL. 2002. Pymol: an open-source molecular graphics tool, p 82–92. In CCP4 newsletter on protein crystallography. Vol. 40.

[59] Pettersen EF, Goddard TD, Huang CC, Couch GS, Greenblatt DM, Meng EC, Ferrin TE. 2004. UCSF Chimera—A visualization system for exploratory research and analysis. J Comput Chem 25:1605–1612. doi:10.1002/jcc.20084 15264254

[60] Eberhardt J, Santos-Martins D, Tillack AF, Forli S. 2021. AutoDock Vina 1.2.0: new docking methods, expanded force field, and python bindings. J Chem Inf Model 61:3891–3898. doi:10.1021/acs.jcim.1c00203 34278794 PMC10683950

[61] Hunter AD. 1997. ACD/ChemSketch 1.0 (freeware); ACD/ChemSketch 2.0 and its Tautomers, Dictionary, and 3D Plug-ins; ACD/HNMR 2.0; ACD/CNMR 2.0. J Chem Educ 74:905. doi:10.1021/ed074p905

